# Identification of 'Candidatus *Neoehrlichia mikurensis*' and *Anaplasma *species in wildlife from Croatia

**DOI:** 10.1186/1756-3305-7-S1-O28

**Published:** 2014-04-01

**Authors:** Relja Beck, Vlatka Čubrić Čurik, Račić Ivana, Šprem Nikica, Vujnović Anja

**Affiliations:** 1 Croatian Veterinary Institute, Savska cesta 143,HR-10 000 Zagreb, Croatia; 2 Faculty of Agriculture, Svetošimunska 25, 10 000 Zagreb, Croatia

## 

Vector borne diseases are classical emerging infectious diseases in human and animal populations. Ticks represent perfect vectors for number of bacteria, parasites and viruses. The genera *Anaplasma*, *Ehrlichia *and recently specified cluster 'Candidatus *Neoehrlichia' *comprise all bacteria from family *Anaplasmataceae*, transmitted by ixodid ticks to mammalian hosts causing infection in humans and animals. 'Candidatus *Neoehrlichia mikurensis*' is one of emrgening vector borne zoonosis recently recognized as human pathogen. First case of human infection was reported from Sweden in 2010 and up to now six human infections were described in Czech Republic, Germany and Switzerland. In 2011 first case of septicaemia in two dogs was reported indicating ability of *Neoehrlichia *to infect various mammalian species, while different rodent species may act as reservoir host. The aim of this study was to identify species from family *Anaplasmataceae *from different species of wild animals (851). The presence of selected pathogens from wild cervids, wild boars, small rodents, muflons, chamois, martens, bears, badgers, wolves, jackals and foxes were determined by performing PCR on spleen samples and subsequent sequencing of fragment of 16S rRNA gene. Sequence analysis revealed presence of *N. mikurensis*, two strains of *Anaplasma phagocytophilum*, *Anaplasma centrale, A. bovis *and *Ehrlichia *sp. Small rodents were infected mainly with *N. mikurensis *together with *A. phagocytophilum*. *Neoehrlichia mikurensis *was single species detected in wild boars, bears and badgers, which represent first finding in these mammalian species. In chamois all species have been found except *A. bovis*. Wild cervids were harbouring both strains of *A. phagocytophilum*, while muflons were infected with *A. centrale, A. phagocytophilum, A. bovis *and *N. mikurensis*. Martens and jackals were free from pathogens while sequencing failed in wolf samples. Foxes were infected with both strains of *A. phagocytophilum *and interestingly in single animal *A. bovis *was confirmed. Two isolates *Ehrlichia *sp. from roe deer and chamois were identical to *Ehrlichia *sp. from Sika deer from Japan. Data obtained from this study clearly present presence of two zoonotic pathogens: *A. phagocytophilum*, causative agent of human granulocytic anaplasmosis (HGA) and newly recognized pathogen *N. mikurensis *in various species. These results also imply importance of wildlife as potential reservoir for ticks and potential transmission to humans.

